# Supplementary material for: Splice-Junction-Based Mapping of Alternative Isoforms in the Human Proteome
Source: Cell Rep. Author manuscript; Available in PMC 2020 Jan 15. (PMC6961840; doi:10.1016/j.celrep.2019.11.026)
Supplement: 2 [file NIHMS1546469-supplement-2.zip › DF1/PXD006675/LeftAtrium/LeftAtrium_46_TPM1_AISEELDHALNDMTSGPMLK.pdf]

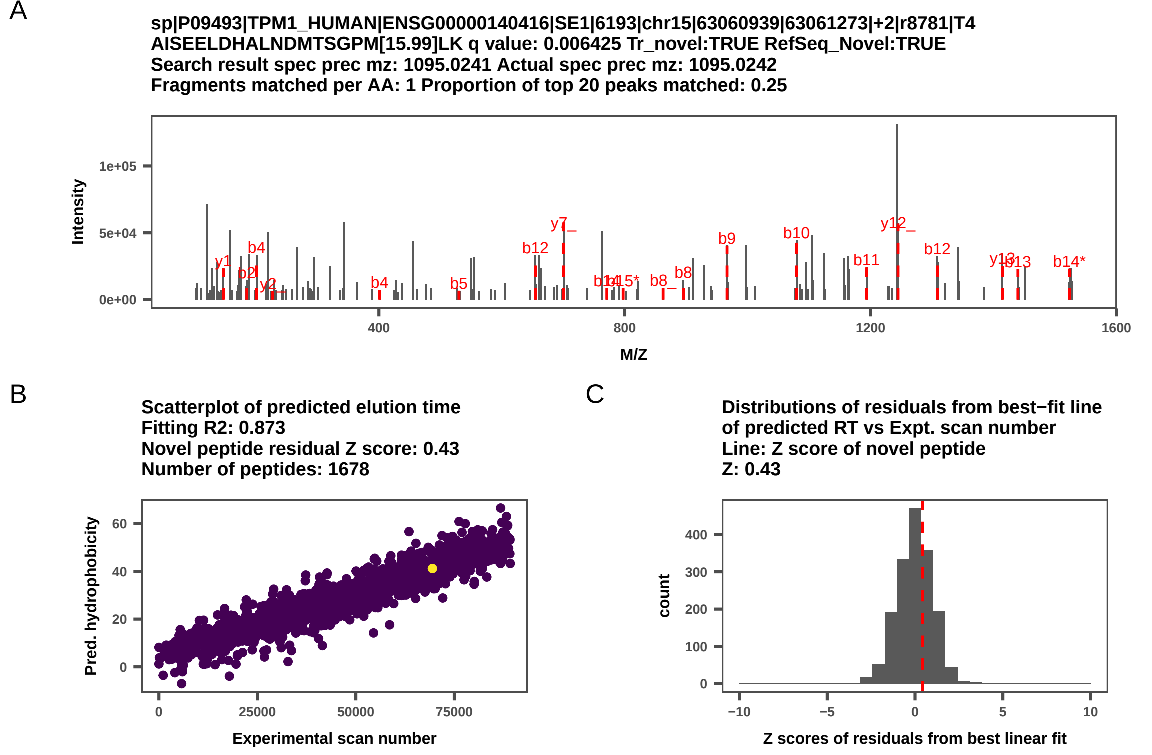
A
sp|P09493|TPM1_HUMAN|ENSG00000140416|SE1|6193|chr15|63060939|63061273|+2|r8781|T4
AISEELDHALNDMTSGPM[15.99]LK q value: 0.006425 Tr_novel:TRUE RefSeq_Novel:TRUE
Search result spec prec mz: 1095.0241 Actual spec prec mz: 1095.0242
Fragments matched per AA: 1 Proportion of top 20 peaks matched: 0.25
Intensity
1e+05
5e+04
0e+00
400
800
1200
1600
M/Z
y1
b4
b2
y2
b4
b5
b12
y7_
b8_
b8
b9
b10
b11
y12_
b12
b13
b14*
B
Scatterplot of predicted elution time
Fitting R2: 0.873
Novel peptide residual Z score: 0.43
Number of peptides: 1678
Pred. hydrophobicity
60
40
20
0
0
25000
50000
75000
Experimental scan number
C
Distributions of residuals from best−fit line
of predicted RT vs Expt. scan number
Line: Z score of novel peptide
Z: 0.43
count
400
300
200
100
0
−10
−5
0
5
10
Z scores of residuals from best linear fit
